# Design and rationale for the non-interventional Global Investigation of therapeutic DEcisions in hepatocellular carcinoma and Of its treatment with sorafeNib (GIDEON) study

**DOI:** 10.1111/j.1742-1241.2010.02414.x

**Published:** 2010-07

**Authors:** R Lencioni, J Marrero, A Venook, S-L Ye, M Kudo

**Affiliations:** 1 Division of Diagnostic Imaging and Intervention, Department of Liver Transplantation, Hepatology and Infectious Diseases, Pisa University School of MedicinePisa, Italy; 2 Multidisciplinary Liver Tumor Clinic, University of MichiganAnn Arbor, MI, USA; 3 University of CaliforniaSan Francisco, CA, USA; 4 Liver Cancer Institute, Zhongshan Hospital, Fudan UniversityShanghai, China; 5 Department of Gastroenterology and Hepatology, Kinki University School of MedicineOsaka, Japan

## Abstract

**Background:**

Hepatocellular carcinoma (HCC) is a complicated condition influenced by multiple confounding factors, making optimum patient management extremely challenging. Ethnicity, stage at diagnosis, comorbidities and tumour morphology affect outcomes and vary from region to region, and there is no common language to assess patient prognosis and make treatment recommendations. Despite recent efforts to reduce the incidence of HCC, most patients present with unresectable disease. Non-surgical treatments include ablation, transarterial chemoembolisation and the multikinase inhibitor, sorafenib, but their effects in all patient subgroups are not known and further information is needed to optimise the use of these treatments.

**Aims:**

The Global Investigation of Therapeutic DEcisions in Hepatocellular Carcinoma and Of its Treatment with SorafeNib (GIDEON) study (ClinicalTrials.gov identifier NCT00812175; http://clinicaltrials.gov/) is an ongoing global, prospective, non-interventional study of patients with unresectable HCC who are eligible for systemic therapy and for whom the decision has been taken to treat with sorafenib under real-life practice conditions. The aim of this study is to evaluate the safety and efficacy of sorafenib in different subgroups, especially Child-Pugh B where data are limited.

**Discussion:**

This study will recruit 3000 patients from > 40 countries and follow them for approximately 5 years to compile a large and robust database of information that will be used to analyse local, regional and global differences in baseline characteristics, disease aetiology, treatment practice patterns and treatment outcomes, with a view to improve the knowledge base used to guide physician treatment decisions and to improve patient outcomes.

**What's known**

- HCC is a complex disease influenced by multiple confounding factors that vary from region to region, making optimum patient management extremely complex.
- Sorafenib is an oral multikinase inhibitor with proven efficacy in patients with unresectable HCC, but data in Child-Pugh B are limited.
- There is a need to fully evaluate existing treatments in all patient subgroups to optimise their use.

**What's new**

- GIDEON will generate data from 3000 patients to evaluate the effects of sorafenib in different patient subgroups, and the resulting large database will be used to analyse local, regional and global differences that influence patient prognosis and management, with a view to refine HCC staging and evaluation and better inform treatment decisions

## Introduction

Hepatocellular carcinoma (HCC) is the sixth most common cancer worldwide, but because of the poor prognosis associated with this disease, it is the third most common cause of cancer-related death (1). Over 80% of patients with HCC are in developing countries, with particularly high incidence rates in sub-Saharan Africa and Southeast Asia (1). There is a low incidence of HCC in developed countries such as the USA, Australia and the UK, but these rates are rising (2).

Risk factors for the development of HCC have been well documented and include the presence of cirrhosis, infection with hepatitis B and C viruses, heavy alcohol intake, diabetes and obesity (1,2). Although surveillance and vaccination programmes have reduced the incidence of HCC in certain populations (3,4), the majority of patients still present with unresectable disease and are unsuitable for surgery. Current treatments for unresectable disease include loco-regional interventions and systemic therapies, although further data on all treatments are required to fully understand their potential, e.g. in patient groups not included in clinical trials.

Non-surgical loco-regional treatment options include ablation therapy and transarterial chemoembolisation (TACE). Ablation therapy is associated with a 5-year survival rate of 40–70%, with best responses seen among patients with single tumours and preserved liver function (5). However, TACE is recommended for patients with large/multifocal tumours with no vascular invasion or extra-hepatic spread and is associated with objective response rates of 16–60% (3). Although survival benefits have been reported in only two randomised controlled trials (RCTs) (6,7), a robust meta analysis showed that the treatment with TACE was associated with significant improvements in 2-year survival vs. control (8). However, TACE treatment has a number of important limitations. Residual tumour growth following treatment means that treatment repetition is necessary. Additionally, many of the clinical studies investigating TACE have used a wide range of treatment strategies, including different types of embolic particle, chemotherapy, emulsifying agent and numbers of treatment sessions. For this reason, there is no clear evidence to support an optimum treatment strategy (9). Also, TACE therapy is only possible in patients where the arterial blood supply to the tumour can be isolated, and is not recommended in patients with portal vein thrombosis, those with Child-Pugh C liver function or those with a total serum bilirubin level > 3 mg/ml, as all of these factors have been identified as predictors of poor prognosis in patients treated with TACE (9). Finally, treatment with TACE is associated with considerable side effects; the most commonly reported being postembolisation syndrome that occurs in > 50% of treated patients (3). Other less frequent but more serious complications include hepatic abscess and cholecystitis. Further research is therefore needed to optimise TACE treatment strategy and ensure treatment efforts are directed at patients who will most likely benefit.

Systemic therapies investigated for unresectable HCC have included single-agent and combination chemotherapy regimens, but their efficacy has been disappointing and their use is no longer recommended (9). More recently, the Sorafenib HCC Assessment Randomized Protocol trial, a multicentre, Phase III, double-blind, placebo-controlled trial of 602 Western patients with unresectable HCC, showed that treatment with the oral multikinase inhibitor, sorafenib (Nexavar®; Onyx Pharmaceuticals, Inc., Emeryville, CA, USA; Bayer HealthCare Pharmaceuticals, Inc., Wayne, NJ, USA; Bayer Schering Pharma AG, Berlin, Germany), was associated with a significant improvement in survival compared with placebo [median overall survival (OS) of 10.7 months vs. 7.9 months for sorafenib and placebo respectively, p < 0.001] (10). As a result, sorafenib is the first systemic anticancer therapy indicated for treating these patients (9). Similar benefits (median OS of 6.5 months vs. 4.2 months for sorafenib and placebo respectively, p = 0.014) were reported in a Phase III, randomised, double-blind, placebo-controlled trial of 226 patients with unresectable HCC from the Asia-Pacific region, thus confirming the efficacy of sorafenib in a broad geographic patient population (11). However, all patients included in these two large RCTs had preserved liver function (Child-Pugh A), and our knowledge regarding the efficacy of sorafenib in patients with hepatic impairment is limited to small subgroups of patients from Phase I and II studies (12,13). Further studies to evaluate the efficacy of sorafenib among all patient groups are therefore needed.

Current treatment guidelines for unresectable disease are therefore based on the best available evidence, including non-randomised trials, case studies and expert opinion; however, significant data gaps exist. Further evidence is needed to fully evaluate current treatment options and optimise their use to improve patient outcomes.

Against this background, the Global Investigation of Therapeutic DEcisions in Hepatocellular Carcinoma and Of its Treatment with SorafeNib (GIDEON) study is an ongoing global, non-interventional study (NIS) of patients with unresectable HCC who are to receive sorafenib as part of their standard clinical care. The study should produce the largest, most robust database of information on factors influencing treatment and outcome of patients with HCC. This manuscript will include details of the GIDEON aims and objectives, study design, target recruitment and timeline. It will also describe the planned analyses and discuss how it is hoped that findings from this study will allow us to gain a detailed understanding of the factors that influence the prognosis and management of these patients, and how this in turn will help us to refine HCC staging and evaluation, better inform treatment choices and ultimately improve outcomes for patients with HCC.

## The GIDEON study

### Aims, objectives and rationale for GIDEON

Non-interventional studies, or observational studies, are postauthorisation safety studies (PASS) that are usually conducted to gain further information about a licensed product. Observational studies are characterised by the fact that assignment to a particular therapy strategy is not mandated by a study protocol but reflects the participating physician’s current practice. The physician alone decides which treatment, if any, is appropriate. In NIS, the decision to include a patient in a study is separate from the treatment decision. Furthermore, no additional diagnostic or monitoring interventions are mandated for the patient as a result of inclusion in an NIS. NIS serve a wide range of purposes but are of particular value in providing information in wider populations or subgroups not covered in RCTs (14). NIS also enable information to be gathered on other parameters not usually assessed in the clinical trial setting, such as patient acceptance and compliance, physician adherence to information and directions for use and prescription behaviour. The importance of NIS in the literature is increasingly recognised, and guidelines were developed to improve the analysis and reporting of observational studies (15). NIS provide opportunities to enhance the evidence base for established drugs and therapies and increase understanding of the impact of treatments in real-world practice (14).

GIDEON is a global, prospective NIS of patients with unresectable HCC who are candidates for systemic therapy and for whom the decision has been taken to treat with sorafenib. It was initiated to fulfil the postapproval commitment to organisations such as the European Medicines Agency to gather data on the safety and efficacy of sorafenib in patients with Child-Pugh B liver function. Additional goals are to compile a large and robust database of HCC treatment patterns and outcomes among patients with unresectable disease who are candidates for systemic therapy, to answer clinically relevant questions and gain a better understanding of the safety of sorafenib with loco-regional therapies, given either concomitantly or sequentially. Data will also be gathered in the USA and possibly other regions on the characteristics, disease course and treatment outcomes of patients with newly diagnosed HCC or recurring disease after curative treatments, who are not candidates for systemic therapy with sorafenib.

Based on these goals, the primary objective of GIDEON is to evaluate the safety of sorafenib in patients with unresectable HCC in real-life practice conditions. Secondary objectives are to: evaluate the efficacy [OS, progression-free survival (PFS), time to progression (TTP), response rate and stable disease rate] of sorafenib in these patients; determine the duration of therapy according to various patient characteristics; evaluate methods of patient evaluation, diagnosis and follow-up; assess comorbidities and their influence on treatment and outcome in real-life practice rather than a controlled clinical trial setting and evaluate the practice patterns of the physicians involved in the care of these patients.

This study was conducted according to established regulations and recommendations relating to the conduct of NIS; volume 9A of the Rules Governing Medicinal Products in the European Union (16). When required, documented approval from the appropriate ethics committee(s)/institutional review board was obtained for all participating centres prior to the study, according to Good Clinical Practice and local laws, regulations and organisations.

### Establishing a global NIS

**Figure 1:**
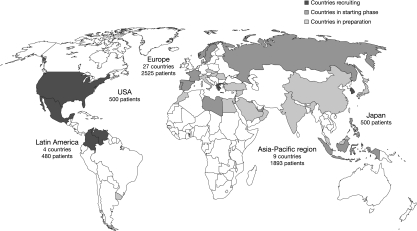
The global reach of GIDEON

GIDEON is a Phase IV, international, prospective, open-label, multicentre, non-interventional PASS of patients with unresectable HCC receiving sorafenib under real-life conditions. Approximately, 3000 eligible patients will be recruited by participating physicians from > 40 countries across Europe, Latin America and the Asia-Pacific region and from the USA (Figure 1) and will be observed from the start of sorafenib therapy to patient withdrawal, loss to follow-up, death or final visit.

**Figure 2:**
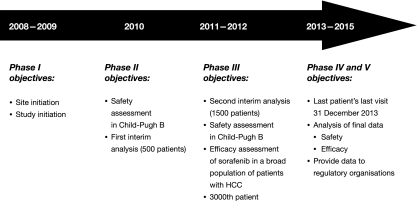
An overview of the GIDEON timeline and planned analyses

An overview of the GIDEON timeline and planned analyses is shown in Figure 2. The first patient’s first visit was recorded in January 2009 and the last patient’s first visit is anticipated to occur in quarter four of 2012. Interim analyses for safety will be conducted after 500 and 1500 patients have been recruited and followed for 4 months, with the final analysis anticipated in quarter two or three of 2014. The study will end 12 months after enrolment of the 3000th eligible patient, irrespective of whether the final patient dies or not, is lost to follow-up or survives.

### Patients to be included in GIDEON

Patients with histologically or cytologically documented or radiographically diagnosed unresectable HCC who are candidates for systemic therapy, and for whom a decision has been made to treat with sorafenib, are eligible for inclusion in GIDEON if they have a life expectancy of > 8 weeks and have provided signed informed consent. Patient exclusion criteria are based on the approved local product information for sorafenib.

### Data collection

**Figure 3:**
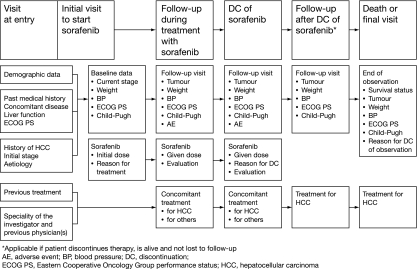
GIDEON patient assessment schedule

**Figure 4:**
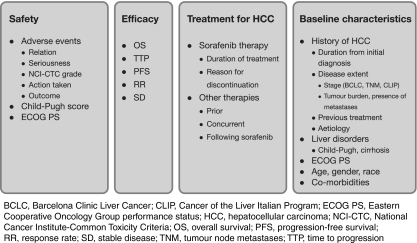
Overview of the GIDEON safety, efficacy, treatment and baseline patient assessments and end-points

All data will be collected using case report forms (CRFs). These will be available as paper and electronic versions, with participating countries and their sites able to choose the format of preference. Data will be collected from all enrolled patients at study entry and start of sorafenib, then at intervals normally used by the prescribing physician (estimation: ≥ 6 to ≤ 12 weeks), or until patient death, withdrawal or loss to follow-up, or if significant changes in a patient’s disease are observed. All data will be verified through spot site monitoring, which will take place at up to 10% of all sites involved in the study. An overview of the visit schedule and data collected at each visit is shown in Figure 3. Study end-points are summarised in Figure 4. All adverse events (AEs) will be graded according to the National Cancer Institute Common Terminology Criteria version 3.0 (National Cancer Institute, Bethesda, MD, USA), and their likely relationship to sorafenib therapy will be documented. Tumour assessments will be made by computed tomography or other equivalent radiographical method and will be evaluated using the Response Evaluation Criteria in Solid Tumors.

The population of patients who entered this study and received at least one dose of sorafenib will be valid for intent-to-treat safety and efficacy analysis. However, patients who received sorafenib in the past will be excluded from efficacy analysis. All baseline demographic data will be summarised for the intent-to-treat population. AEs and other safety parameters, including blood pressure, Child-Pugh grade and Eastern Cooperative Oncology Group performance status (ECOG PS), will be summarised using the safety population.

Planned subgroup analyses conducted globally, regionally and by country will include: the impact of baseline characteristics on safety, particularly Child-Pugh B; the relationship between baseline characteristics and efficacy; the duration of sorafenib therapy and reasons for discontinuation; the effect of other treatments for HCC on outcome and the impact of different practice patterns on outcome. In addition, subgroup analyses for specific regions may be conducted, such as: an evaluation of common treatments for HCC in Asia; referral and diagnostic patterns in Europe; duration of treatment, tolerability and compliance in Latin America and patient selection for loco-regional therapy in Japan. However, all subgroup analyses performed will depend on the actual data collected.

### Statistical considerations for GIDEON

An overall sample size of 3000 patients with unresectable HCC treated with sorafenib is expected to be sufficient to allow for evaluation of safety of the overall population as well as specific subgroups. With this sample size, there would be an 84% chance of observing an AE with a true incidence of 1% in at least 25 patients.

All baseline, safety and efficacy data were analysed using descriptive statistics. Kaplan–Meier estimates were calculated for the OS, PFS and TTP efficacy end-points. At the time of the analyses, any patient alive or lost to follow-up, without disease progression or death or without documented radiological progression will be censored at the last date of evaluation for OS, PFS and TTP analyses, respectively. Exploratory subgroup analyses of efficacy and safety data may also be performed, stratified by prognostic/predictive baseline factors such as stage, Child-Pugh score, ECOG PS, region and age, as appropriate. Data regarding administration of sorafenib such as duration, given dose, continuation or discontinuation of therapy and dose modification of sorafenib therapy, including reason(s) for discontinuation, will be summarised in a descriptive manner. Treatments for HCC other than sorafenib before, during and after therapy with sorafenib will be summarised descriptively as per available data. The sample size was calculated to collect data to allow for sufficient evaluation of safety monitoring of all treated patients. Interim analyses are planned during the study primarily for summarising and monitoring safety data, and will be conducted after 500 patients and 1500 patients are enrolled and have been treated on study for at least 4 months.

## Discussion

### What will GIDEON achieve?

GIDEON is a global PASS initiated to collect more information on the safety and efficacy of sorafenib in patients with unresectable HCC. This is the largest prospective HCC NIS in the world, which will enrol 3000 patients from > 40 countries globally. The compilation of a database of this size in HCC has not previously been undertaken; as no other global registries exist in this area, it is anticipated that the data collected here will be an important contribution to our knowledge and will help to answer important and clinically relevant questions relating to the natural disease course of HCC and liver dysfunction, long-term efficacy and safety of sorafenib therapy, physicians’ practice patterns and patients’ perspectives.

The number of factors influencing HCC and its disease course make it extremely difficult to accurately assess patient prognosis and make optimum treatment recommendations. The geographical variation of these factors has also prevented the establishment of a universal system to assess all patients. Findings from GIDEON could help to establish a globally applicable staging classification, which could facilitate the accurate and consistent assessment of all patients and help to provide a common language for the broad HCC multidisciplinary team on which to base treatment recommendations. In addition, as GIDEON will collect data regarding the differences in physician treatment practice patterns and outcomes, it may be possible to analyse these data with a view to optimise the role of all members of this large multidisciplinary team and streamline patient care.

Although potentially curative therapy via surgical resection or transplant is possible for some patients with HCC, there is a lack of cadaveric transplants available, and the majority of patients are unsuitable for surgery at presentation. In addition, the recurrence rate of HCC after curative treatment is high and the long-term curable rate is low (17). Thus, non-surgical treatments play a central role in the management of these patients. However, there is still a relative shortage of RCTs to fully evaluate these treatments in all patient subgroups, and more work is needed to fully understand the benefits of each of these treatments and to establish their place in the HCC treatment armamentarium. One of the main focuses of GIDEON is to gain further information on the optimum duration of sorafenib therapy as well as the safety and efficacy of sorafenib in different subgroups of patients, especially patients who are generally excluded from RCTs to minimise errors or confounding factors in the study, i.e. patients who have moderate liver dysfunction (Child-Pugh B) where data are currently limited. However, information is also being gathered regarding the safety and outcomes following other treatments before, during and after sorafenib therapy; thus, it is anticipated that the information gathered in this study will help to improve our understanding of the risks and benefits associated with each of these treatment approaches, which could help us to establish an optimum treatment algorithm for these patients.

In addition to GIDEON, a large clinical trial programme for sorafenib is ongoing that should help us to fully establish its optimum place in therapy. One area of interest is the role of sorafenib as adjuvant therapy to improve survival of patients with HCC. To date, treatments such as radiotherapy and chemotherapy and their combination have been used to reduce tumour size and improve patients’ quality of life (18). TACE has recently been shown to be the only palliative treatment that can benefit HCC patients ineligible for curative treatments because of advanced tumour stage or poor hepatic functional reserve; however, the survival gain appears marginal (19) and other effective treatments are urgently needed. Against this background, a large Phase III, randomised, double-blind, placebo-controlled trial of adjuvant sorafenib following either surgical resection or local ablation is currently in progress (STORM study) (20). The primary end-point is recurrence-free survival, with secondary end-points including time to recurrence and OS. Estimated accrual to this trial is 1100 patients and data are due to be reported in 2014.

Another area of interest is the efficacy of sorafenib in combination with, and subsequent to, TACE therapy. A large randomised Phase II trial has been initiated to evaluate the role of sorafenib in combination with TACE in the treatment of patients with intermediate disease (SPACE study) (21). Estimated enrolment to this trial is 350 patients, and final results are expected in late 2010. In addition to this, a Phase III, double-blind, randomised, placebo-controlled trial of sorafenib following TACE in Japanese patients with unresectable, advanced disease is ongoing (Japan post-TACE study) (22). The target recruitment of 414 patients has already been reached and final results are anticipated in early 2010.

Finally, the effects of sorafenib in combination with other targeted agents in HCC are also of interest, and a large Phase III, randomised, double-blind trial evaluating the efficacy, safety and health-related quality of life of sorafenib plus erlotinib vs. sorafenib plus placebo for the first-line treatment of advanced HCC is in progress (SEARCH study) (23). The target recruitment for this study is 700 patients and the estimated final data collection date is July 2011.

These ongoing studies form a comprehensive and well-integrated clinical trial programme, which will provide data from several points in the treatment pathway. GIDEON is also a key component of this programme, as it will provide data from the entire unresectable patient population treated under real-life conditions, not in a non-restrictive setting and within the approved indication, and will enable the collection of data from populations not commonly included in RCTs, such as those with Child-Pugh B liver function. Thus, in addition to the ongoing interventional studies, the information collected in GIDEON will significantly contribute to the current body of evidence, which helps inform treatment decisions.

### What are the limitations of GIDEON?

The information gathered in GIDEON will form a large and robust database that will be analysed to improve our understanding of the global, regional and local differences in patient demographics, disease course and treatment outcomes, with a view to improve our knowledge base and improve patient outcomes. However, as this is an observational NIS, it is associated with a number of limitations. The lack of randomisation to specific treatment arms and the lack of a placebo-control arm will limit any robust evaluation of the efficacy of any of the treatments received by these patients during the course of the study. Comparisons between sorafenib-treated and untreated patients in the USA will be limited because of the small sample size of patients in the USA who will not receive sorafenib. The value of some subgroup analyses may also be limited by small patient numbers, although these analyses may still be hypothesis-generating and could help direct future research. However, given these limitations, it will be important to consider findings from GIDEON together with emerging data from large RCTs, to fully evaluate the safety and efficacy of these treatments and draw any definitive conclusions.

Another possible limitation in GIDEON is that all data will be collected via the completion and submission of CRFs, which could delay data collection and evaluation. However, electronic versions of the CRF have been compiled with a view to facilitate this process and to reduce any lengthy delays in completing and analysing the data collected.

As with any observational study, a number of biases may also exist. The lack of blinding of either the treating physician or the patient to study treatment may introduce a bias in reporting treatment outcomes. It is also possible that physicians engaged in GIDEON may be more likely to choose sorafenib therapy for a larger proportion of their patients than would be representative of normal treatment practice patterns in that area, although all patients receiving sorafenib therapy must be eligible according to the locally approved product information for sorafenib. Finally, while study procedures regarding data collection and verification are in place for GIDEON, it should be noted that in an NIS, potential exists for less robust data than might be expected from an RCT.

## Conclusions

GIDEON is the largest global, prospective, open-label NIS ever conducted among patients with unresectable HCC. The study was initiated to further evaluate the safety and efficacy of sorafenib in different patient subgroups, including Child-Pugh B. The collection of information regarding patient baseline demographics, disease aetiology, treatments and outcomes from approximately 3000 patients worldwide, over a period of approximately 5 years, will also enable the compilation of a large and robust database that will be used to analyse local, regional and global differences, with a view to answer some important questions and fill significant data gaps. It is therefore anticipated that findings from GIDEON, together with data from large RCTs, will help improve the knowledge base used to guide physician treatment decisions and may enable physicians to make better informed treatment choices and ultimately improve patient outcomes.
